# Mycobacterial infection induces higher interleukin-1β and dysregulated lung inflammation in mice with defective leukocyte NADPH oxidase

**DOI:** 10.1371/journal.pone.0189453

**Published:** 2017-12-11

**Authors:** Wen-Cheng Chao, Chia-Liang Yen, Cheng-Yuan Hsieh, Ya-Fang Huang, Yau-Lin Tseng, Peter Andrija Nigrovic, Chi-Chang Shieh

**Affiliations:** 1 Institute of Clinical Medicine, National Cheng Kung University Medical College, Tainan, Taiwan; 2 Department of Medical Research, Taichung Veterans General Hospital, Taichung, Taiwan; 3 National Laboratory Animal Center, National Applied Research Laboratories, Tainan, Taiwan; 4 Department of Surgery, Division of Thoracic Surgery, National Cheng Kung University Hospital, Tainan, Taiwan; 5 Division of Rheumatology, Immunology, and Allergy, Brigham and Women’s Hospital, Boston, Massachusetts, United States of America; 6 Division of Immunology, Boston Children’s Hospital, Boston, Massachusetts, United States of America; 7 Department of Pediatrics, National Cheng Kung University Hospital, Tainan, Taiwan; Rutgers Biomedical and Health Sciences, UNITED STATES

## Abstract

Granulomatous inflammation causes severe tissue damage in mycobacterial infection while redox status was reported to be crucial in the granulomatous inflammation. Here, we used a NADPH oxidase 2 (NOX2)-deficient mice (*Ncf1*^*-/-*^) to investigate the role of leukocyte-produced reactive oxygen species (ROS) in mycobacterium-induced granulomatous inflammation. We found poorly controlled mycobacterial proliferation, significant body weight loss, and a high mortality rate after *M*. *marinum* infection in *Ncf1*^*-/-*^ mice. Moreover, we noticed loose and neutrophilic granulomas and higher levels of interleukin (IL)-1β and neutrophil chemokines in *Ncf1*^*-/-*^ mice when compared with those in wild type mice. The lack of ROS led to reduced production of IL-1β in macrophages, whereas neutrophil elastase (NE), an abundant product of neutrophils, may potentially exert increased inflammasome-independent protease activity and lead to higher IL-1β production. Moreover, we showed that the abundant NE and IL-1β were present in the caseous granulomatous inflammation of human TB infection. Importantly, blocking of IL-1β with either a specific antibody or a recombinant IL-1 receptor ameliorated the pulmonary inflammation. These findings revealed a novel role of ROS in the early pathogenesis of neutrophilic granulomatous inflammation and suggested a potential role of IL-1 blocking in the treatment of mycobacterial infection in the lung.

## Introduction

*Mycobacterium tuberculosis* (*M*. *tb*) infection remains one of leading health problems in the world, with 10.4 million new tuberculosis (TB) cases in 2015 [1]. Tissue responses to *M*. *tb* infection are characterized by caseous granulomatous inflammation, consisting of not only macrophages and lymphocytes but also neutrophils [2, 3]. Furthermore, a non-resolving inflammation induced by the complex survival strategies of *M*. *tb* may lead to a dysregulated and host-detrimental inflammation resulting in severe tissue damage, including the formation of pulmonary cavities [4].

Neutrophils are one of the predominant cell types in the airways of patients with active TB [3]. It has been postulated that neutrophils play a pathogenic, rather than protective, role which leads to host-detrimental inflammation [5]. Additionally, pulmonary epithelial cells, the first-line pulmonary cells to be exposed to *M*. *tb* in TB infection, were recently found to secrete CXCL5, a potent neutrophil chemokine, in mycobacterial infections [6]. Moreover, reactive oxygen species, mainly produced through NADPH oxidase 2 (NOX2) by neutrophils in inflammatory tissues, were recently reported to be important for the organization of granulomas [7]. Our previous studies implicated that abnormal ROS production by leukocytes may contribute to the increased severity of mycobacterial infection and lead to more severe pulmonary tuberculosis (TB) in diabetics [8]. These findings implicate a potential role of neutrophils in the early phase of granulomatous inflammation. Nonetheless, how the early recruited neutrophils exert a potentially host-detrimental effect on tissue inflammation through ROS in mycobacterial infection remains elusive.

IL-1β has been shown to play a pivotal role in the anti-TB inflammatory network given that IL-1β has been shown to mediate the cross-talk between cytokine and eicosanoid pathways [9–11]. Our previous studies revealed that ROS regulate the production of IL-1β through modulating the activation of the inflammasome in mycobacterium-infected macrophages [8]. We also identified ROS as the key regulator of IL-1β production in a serum-induced arthritis mouse model through an inflammasome-independent pathway [12]. In that model, an early neutrophilic inflammation was noted in ROS-deficient mice. We therefore postulated that neutrophils may be involved in the linkage between ROS and IL-1β in mycobacterial infection. In this study, we aimed to investigate the role ROS in mycobacterium-induced granulomatous inflammation, focusing on addressing the role of neutrophils and elucidating how ROS regulate IL-1β production in pulmonary infection by mycobacteria.

## Materials and methods

### *Mycobacterium marinum* preparation

Given that Region of Difference-1 (RD-1) locus plays a critical role in TB virulence, we used *M*. *marinum* in this study, which is an RD-1 containing non-tuberculosis mycobacterium. *M*. *marinum*, obtained from American Type Culture Collection (ATCC), was further confirmed using chip hybridization and 16S rRNA sequencing.

### Mouse experiments

Male WT C57BL/6 (B6) mice and NADPH oxidase deficiency (*Ncf1*^*-/-*^) mice were infected with 3x10^7^ *M*. *marinum* in 20 μl phosphate buffered saline (PBS) via intra-tracheal injection (i.t.). To minimize the effects of mycobacterial load on cytokines production, we decreased *M*. *marinum* to 3 x10^6^ in experiments studying the effects of ROS on the production of cytokines and chemokines. p47^phox-/-^ mice with a point mutation in the splice site of exon 8 in *Ncf1*, were purchased from The Jackson Laboratory and were maintained in the Laboratory Animal Center of the National Cheng-Kung University Medical College. For mouse infection, *M*. *marinum* grew to logarithmic growth phase in 7H11-broth and were then collected by centrifugation (2400g, 10 min). Euthanasia of mice was conducted at 7 and 14, days after injection. Pulmonary tissues were embedded in paraffin wax, and the slides were stained by acid-fast stain or hematoxylin and eosin stain for histopathological analysis. Animals were sacrificed by exsanguination under anesthesia. All studies were conducted in accordance with the National Institutes of Health Guide for the Care and Use of Laboratory Animals and were approved by the Institutional Animal Care and Use Committee (IACUC) guidelines of the National Cheng Kung University (No. 102242, 103017, 104156, 105041, and 106029).

### Histopathological examinations and immunostaining

A histopathologic analysis of infected organs was performed in each experiment. Lungs were fixed in 10% formaldehyde and embedded in paraffin for subsequent hematoxylin and eosin (H&E) staining, Ziehl-Neelsen acid-fast staining, immunohistochemical (IHC) staining of F4/80, and Immunofluorescent (IF) staining of both F4/80 and NE. A 3-μm section was taken of the lungs buffered formalin for H&E staining to evaluate airway inflammation and identify granulomas, and a 5-μm section was used in Ziehl-Neelsen acid-fast staining to visualize mycobacteria within granulomas. Lung sections were stained with F4/80 antibody (Abcam, USA), with antigen retrieval of 0.1% trypsin for 120 minutes, to determine locations of macrophages within granulomas. To demonstrate the mosaic pattern of neutrophilic granulomas mixed by neutrophils and macrophages, we used two IF staining in this study, with anti-neutrophil elastase antibody for neutrophils and anti-F4/80 antibodies for macrophages. To test whether abrogation signaling of IL-1β could restore the regulated inflammation, we used rat anti-mouse monoclonal antibody directed against IL-1β (Biolegend, USA) and IL-1 receptor antagonist (Anakinra, Kineret^®^) in this study.

### Enumeration of colony forming units (CFU)

Frozen lungs were homogenized in 3 ml DMEM supplemented with 0.1% Triton-X, using an AHS200 homogenizer, and then was subjected to serial dilutions and plated on 7H10 plates and a number of bacteria presented as CFUs per ml. Given that *M*. *marinum* grows quickly at low temperature and in the dark, we set the culture condition at 32°C and covered plates with foil. The number of viable mycobacteria recovered from frozen lungs was determined after incubation with 5% CO2 for 12~14 days, and the mycobacterial load is presented as CFUs/gram tissue.

### Flow cytometric analysis of neutrophil counts

Lungs were recovered, weighed, incubated in 2 mg/ml collagenase D and 40 U/ml DNase I solution, and were dispersed by passage through a 70 mm mesh. After lysis of red blood cells, viable cells were counted. For immunophenotyping, cells were incubated with fluorescence-conjugated antibodies. Antibodies (BD Pharmingen, USA, California) used are against CD11b, F4/80, and Ly6G.

### Quantification of infiltrative area

To measure the area of granulomatous inflammation with and without a depletion of IL-1β, we captured 4 digital images (Leica DM2500) with inflammation along with bronchioles in each mouse and determined the infiltrative area by *Image J software* at 20x magnification. Results were expressed as a percentage of the infiltrative area divided by the total lung area.

### Protein analysis and Western blotting

For the detection of pro-IL1β (p34) and mature IL-1β (p17), the blot was probed with 1:1000 rabbit anti-human IL-1β antibody (Santa Cruz Biotechnology, USA, California) and cleaved IL-1β antibody (Cell Signaling, USA), respectively.

### Cytokine and chemokine measurement

Cytokines and chemokines were determined by ELISA (IL-1β and IL-10, Biolegend, San Diego, USA; CXCL5, R&D Systems, Minneapolis, USA; CXCL1 and CCL5, PeproTech, Rehovot, Israel; IL-6, TNF-α, IFN-γ, and IL-17A, ebioscience, San Diego, USA). NE activity was measured by Neutrophil Elastase Activity Assay Kit (Cayman, USA).

### Cells preparation

We injected 2.5 ml of 3% thioglycollate medium into the peritoneum, and harvest peritoneal cells at 4 hours as neutrophils and at 72 hours as macrophages. THP-1 cells were treated used 100 nM PMA for 24 hr as differentiation condition into macrophages in this study. Human granulocytes were isolated through 6% dextran for 2hr for RBCs sedimentation and the centrifugation (400g for 20 minutes) in Ficoll-Hypaque (Pharmacia, Uppsala, Sweden)

### Statistical analysis

Data were presented as percentages for categorical variables and as means ± standard deviations for continuous variables. Differences between two subgroups were analyzed by the Mann-Whitney U test. The difference of survival between two groups was determined by Log-rank test, and the correlation between two continuous variables was analyzed by Spearman’s rank correlation analysis. Statistical significance was set at *P*<0.05, two-sided. Data were analyzed using Prism version 5.0.

## Results

### Poorly controlled mycobacterial load, significant body weight loss, and high mortality in *M*. *marinum*-infected *Ncf1*^*-/-*^ mice

**Fig 1:**
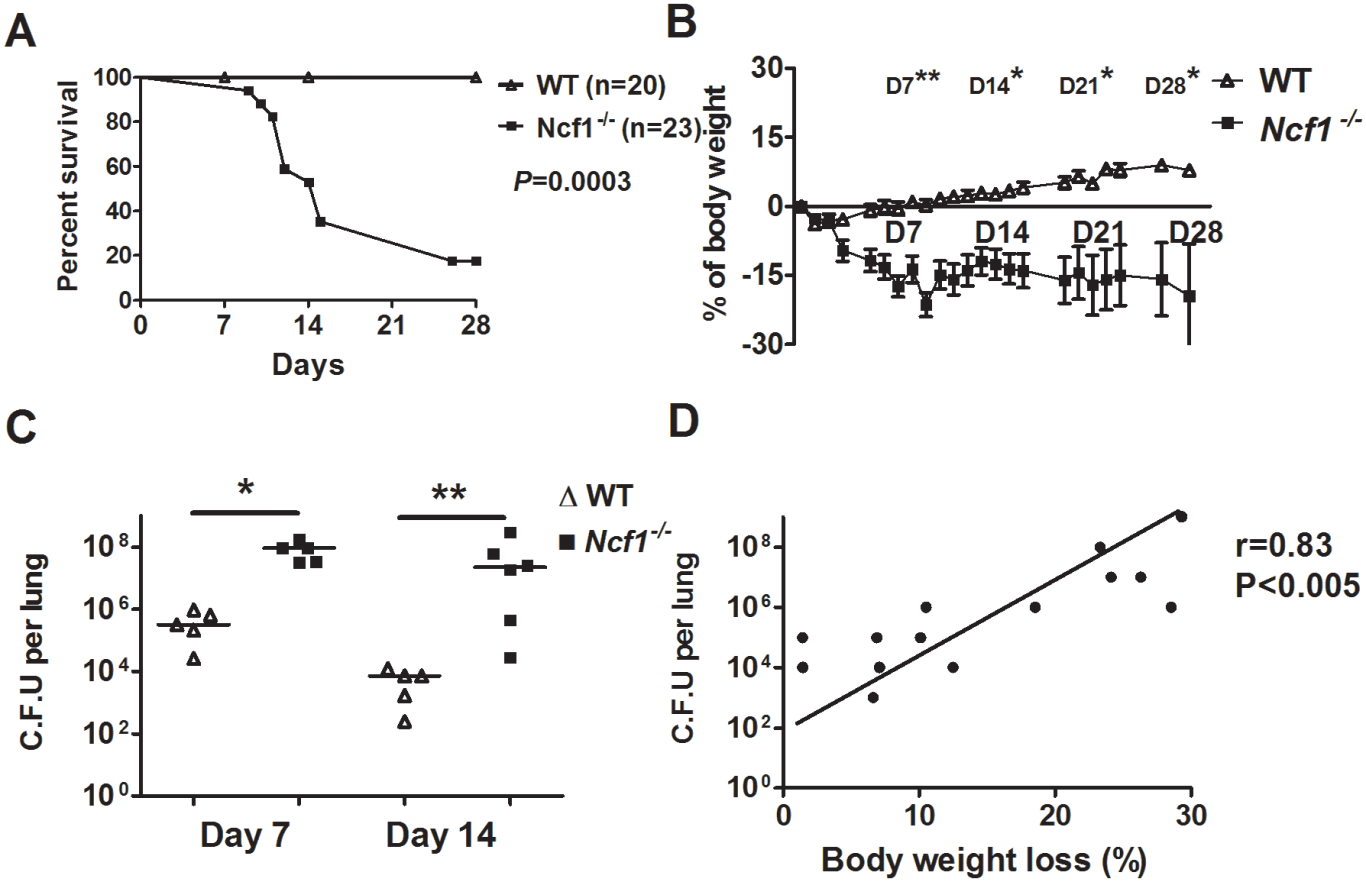
Increased severity and mortality of *M*. *marinum* pulmonary infection in *Ncf1*^*-/-*^ mice. *Ncf1*^-/-^ (loss of function mutation in p47phox) and WT controls were intra-tracheal injected with *M*. *marinum* (3 x 10^7^ CFU). Survival (A) and changes in body weight (B) were monitored over the 28 days period following *M*. *marinum* infection in WT (n = 20) and *Ncf1*^-/-^ mice (n = 23). The number of viable mycobacteria (C) was determined at 7 days and 14 days after *M*. *marinum* infection. Data are shown as a mean log of CFU per paired-lung (5 mice per group). The high correlation between changes in body weight and a number of viable mycobacteria in lungs was demonstrated in (D). Data represented mean ± sd. The experiments were analyzed with Log-rank test (A), Kruskal-Wallis test (C), and Spearman’s rank correlation analysis (D) *p < 0.05; **p < 0.005. These experiments were repeated twice with similar results.

Wild-type BL6 (WT) mice and *Ncf1*^*-/-*^ mice, which are deficient in the gene encoding p46phox of phagocytic NADPH oxidase (NOX2), were first injected with *M*. *marinum* (3 x 10^7^ CFU) via the trachea. WT mice apparently were more resistant to the infection during a 4-week observation. In contrast, *Ncf1*^-/-^mice showed early mortality with an approximate death rate of 60% during the period between the 7^th^ day and 14^th^ day after the *M*. *marinum* infection (Fig 1A). The high mortality of *Ncf1*^*-/-*^ mice was associated with an abrupt and significant weight loss after infection while the body weight was stable in WT mice (Fig 1B). To determine the control of mycobacterial infection in WT and *Ncf1*^*-/-*^ mice, we measured the colony-forming unit (CFU) of *M*. *marinum* in the whole lung homogenates and found that WT mice gradually controlled mycobacterial growth, whereas the mycobacteria continued to grow in *Ncf1*^-/-^mice (Fig 1C). Moreover, we found a high degree of association between percentage of body weight loss and the mycobacterial load in both species of mice (*r* = 0.83, *P*<0.0001) (Fig 1D) (See detailed individual data in S1 Dataset). Collectively, these results indicated that the immune defense of *Ncf1*^*-/-*^ mice infected with *M*. *marinum* is not effective in controlling the growth and invasion of the mycobacteria in the lung.

### Loose cell aggregations with neutrophilic infiltration in *Ncf1*^-/-^ mice in comparison with compact and organized granulomas in WT mice

**Fig 2:**
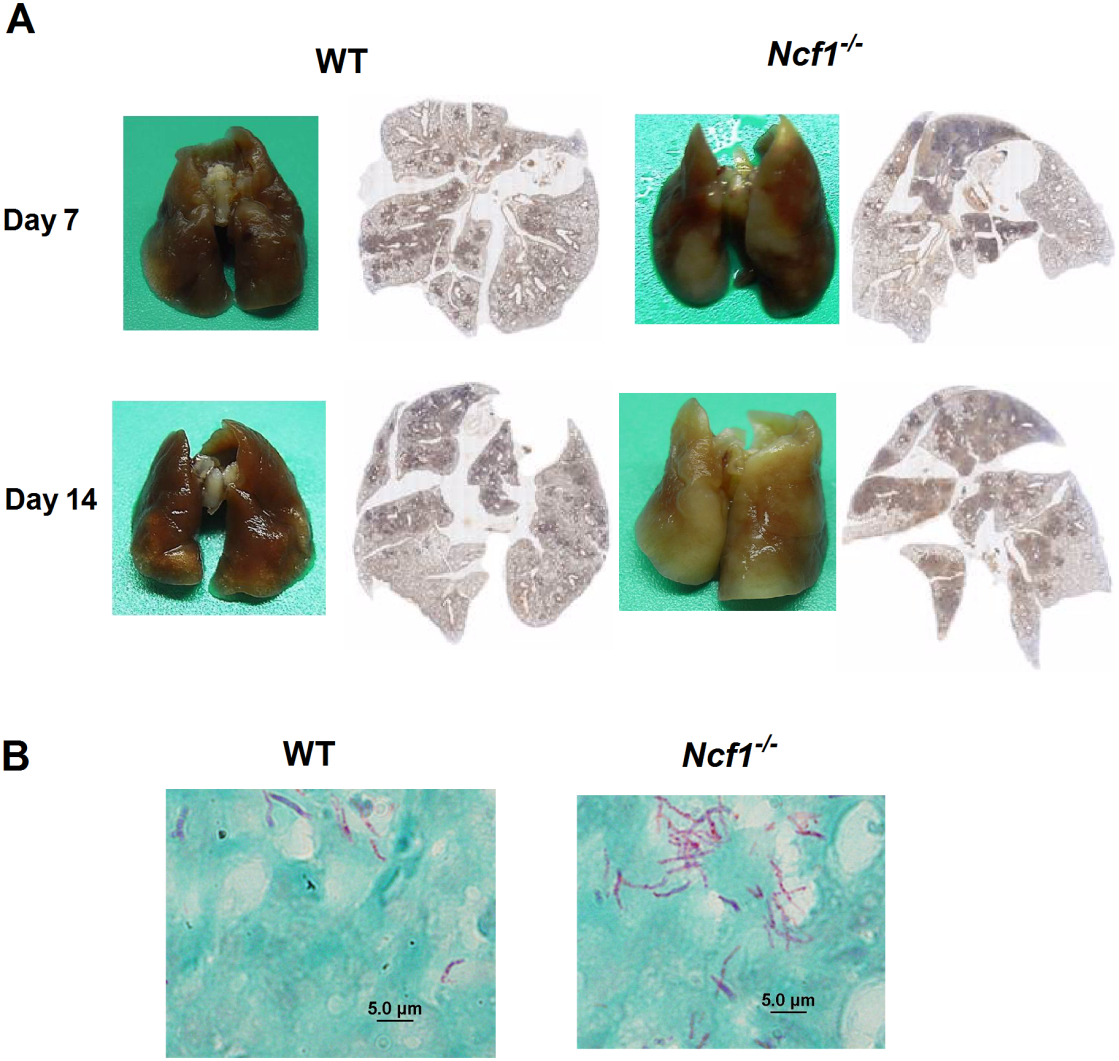
A Higher level of pulmonary inflammation in *Ncf1*^*-/-*^ mice after *M*. *marinum* infection in comparison with the inflammation in WT mice. Representative gross pictures (A) and cross-sectional histological examinations (B) of *M*. *marinum*-infected lungs from WT and *Ncf1*^*-/-*^ mice at day 7 and day 14 after infection. Acid-fast stains (AFS) (B) at day14 showed abundant AFS-positive bacilli in *Ncf1*^-/-^ mice, whereas sparse AFS-positive bacilli were found in WT mice. These experiments were repeated with similar results.

To characterize the role of ROS in mycobacterium-induced granulomatous inflammation, we then went on to examine the pulmonary inflammation in response to *M*. *marinum* infection. The gross appearance of the infected lungs showed a markedly increased pulmonary inflammation in *Ncf1*^*-/-*^ mice. The histological examination of the whole lung cross-section samples further demonstrated an extensive pulmonary infiltration spreading to the whole lung on day 14 in *Ncf1*^*-/-*^ mice, whereas the inflammation appeared tobe localized to the tissues close to the airways in the lungs of WT mice (Fig 2A). The acid-fast stain (AFS), used to stain mycolic acid of mycobacteria, clearly demonstrated the presence of AFS-positive bacilli in the lungs (Fig 2B). Consistent with the data of CFU counts (Fig 1C), AFS-positive bacilli were apparently more abundant in *Ncf1*^-/-^mice when compared with the sparse AFS-positive bacilli in WT mice.

**Fig 3:**
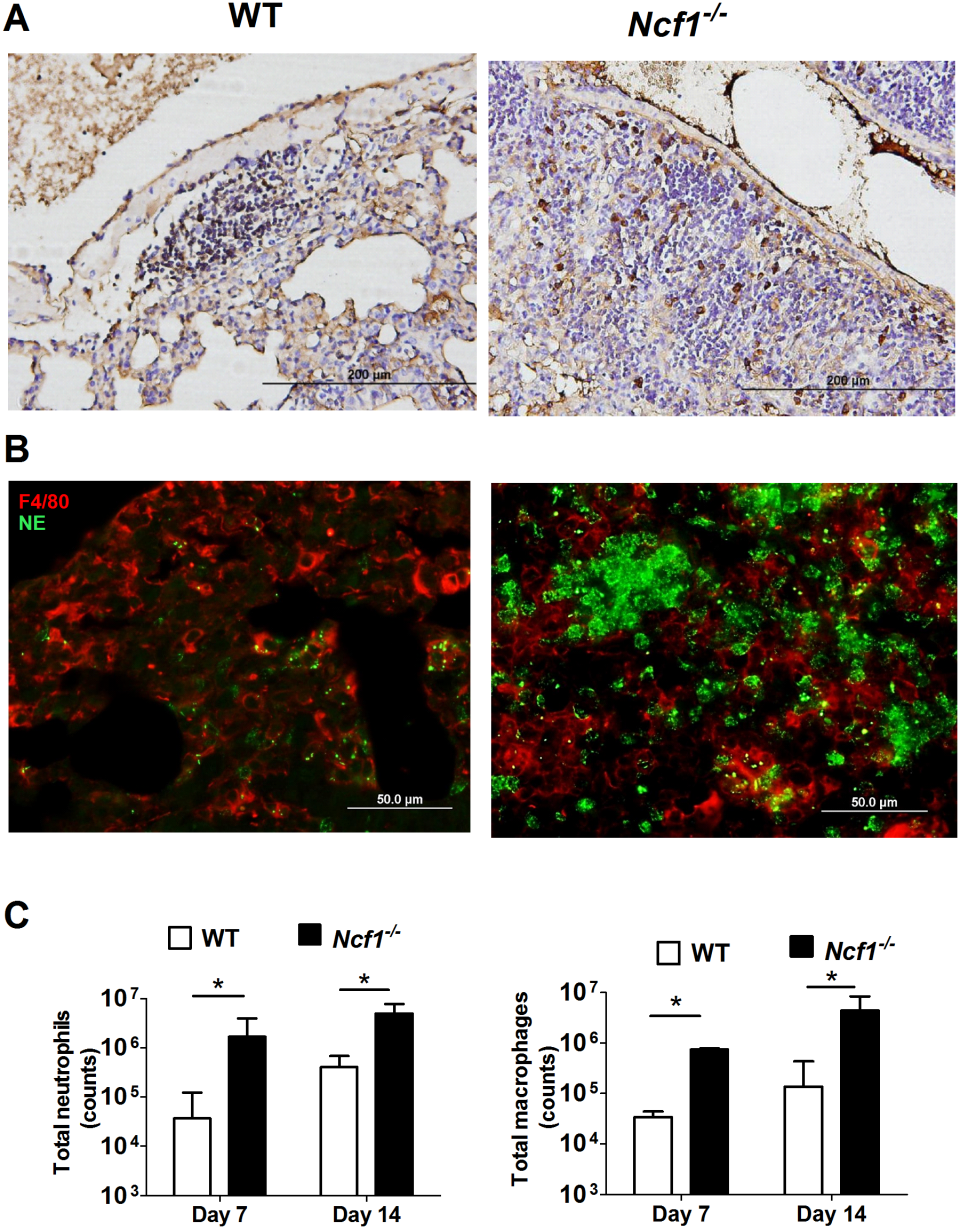
The loose and neutrophilic granulomas in *Ncf1*^-/-^ mice in comparison with the compact granulomas in WT mice. The representative immunohistochemical stain of macrophages (F4/80) (A) showed compact aggregation of macrophages in WT mice, whereas macrophages were scattered within the granuloma of *Ncf1*^-/-^ mice. The immunofluorescent stain (B) (F4/80: red; Neutrophil elastase (NE): green) illustrated much more NE-positive neutrophils interspersed among macrophages in *Ncf1*^-/-^ mice compared with sparse neutrophils in WT mice. (C) Neutrophil and macrophage counts of *M*. *marinum*-infected WT and *Ncf1*^-/-^mice at day 7 and day 14 were analyzed by flow cytometry, while CD11b^+^Ly6G^+^ represented neutrophils and CD11b^+^F4/80^+^ represented macrophages. Data represent mean ± sd of 4 mice from two independent experiments. The experiments were analyzed with Kruskal-Wallis test. *p < 0.05; **p < 0.005. These experiments were repeated twice with similar results.

To characterize the differences in granulomatous inflammation between *Ncf1*^-/-^ mice and WT mice, we used immunohistochemical stain to identify macrophages (F4/80 staining) given that macrophages are the essential cells of mycobacterium-induced granulomas. Contrary to the compact aggregation of macrophages in WT mice, macrophages in *Ncf1*^-/-^ mice were less aggregated, with macrophages scattered within the inflammatory area (Fig 3A). Based on the previous reports that neutrophils were found to be the predominant cells in both human and mouse mycobacterial infection [3, 13], we also investigated the infiltration of neutrophils in the infected lungs. Using immunofluorescent staining (F4/80 for macrophages: red; NE for neutrophils: green), we showed that NE-positive neutrophils interspersed among macrophages in *Ncf1*^-/-^ mice, while only scanty neutrophils were observed in WT mice (Fig 3B). To quantify leukocytes in the excessive inflammation of *Ncf1*^*-/-*^ mice, we used flowcytometry to analyze the single cell suspension from the lung tissues from WT and *Ncf1*^*-/-*^ mice for neutrophils (Cd11b^+^Ly6G^+^) and macrophages (Cd11b^+^F4/80^+^). We found that both neutrophil and macrophage numbers in *Ncf1*^*-/-*^ mice were higher than those in WT mice (both day 7, and day 14; Fig 3C). Taken together, these data showed that ROS-deficiency leads to a disordered inflammation, with an early influx of macrophages and neutrophils, which lead to loose inflammatory cell aggregations.

### High levels of IL-1β and neutrophilic chemokines in *M*. *marinum*-infected *Ncf1*^*-/-*^ mice

**Fig 4:**
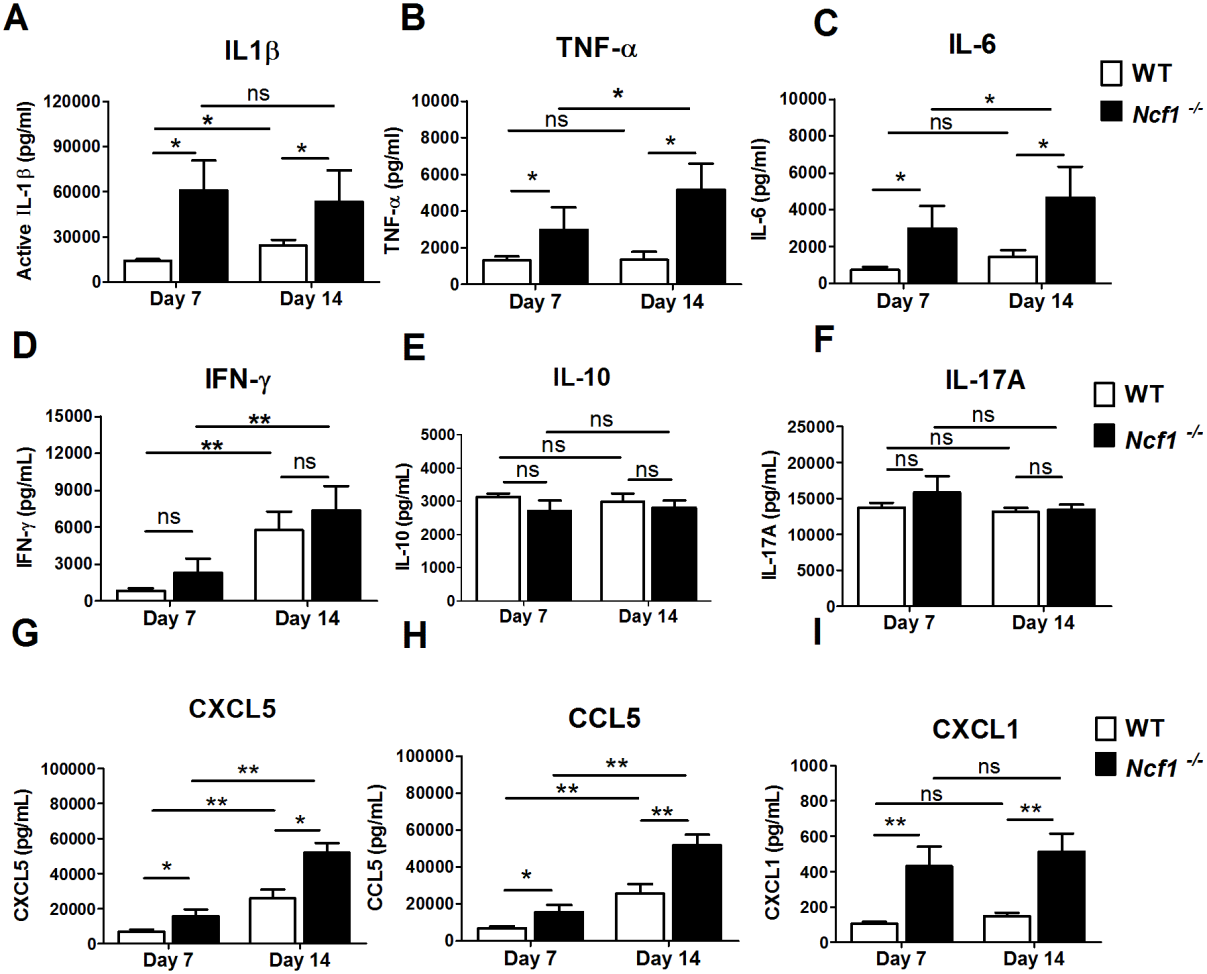
High levels of IL-1β and neutrophilic chemokines in *M*. *marinum*-infected *Ncf1*^-/-^ mice. Cytokine and chemokine responses to *M*. *marinum* infection (3 x 10^6^ CFU). Cytokines of innate immunity including IL-1β (A), TNF-α (B) and IL-6 (C); cytokines of adaptive immunity including IFN-γ (D), IL-10 (E) and IL-17 A(F), and neutrophilc chemokines including CXCL5 (G), CCL5 (H) and CXCL1 (I) were assessed in lung homogenates obtained 7 days and 14 days after *M*. *marinum* infection (*3* x 10^6^ CFU). Data represent mean ± sd (n = 4–7 mice each group) The experiments were analyzed with Kruskal-Wallis test. *p < 0.05; **p < 0.005 and repeated with similar results.

We went onto to characterize the cytokine and chemokine profiles in WT and *Ncf1*^*-/-*^ mice after infected with *M*. *marinum* (3 x 10^6^ CFU). Higher levels of innate immunity-associated cytokines including IL-1β (Fig 4A), TNF-α (Fig 4B) and IL-6 (Fig 4C) in lung homogenates were found in *Ncf1*^*-/-*^ mice when compared with those in WT mice on both day-7 and day-14. Notably, the extent of elevation of IL-1β, a critical innate immunity-associated cytokine, was more marked when compared with the other two cytokines. Additionally, an early elevation of IL-1β was noted in *Ncf1*^*-/-*^ mice with a similar high level of IL-1β on both day7 and day14, whereas levels of TNF-α and IL-6 were higher on day 14 than those on day 7. In contrast to innate immunity-associated cytokines, no difference was found in the adaptive immunity-associated cytokines including IFN-γ (Fig 4D), IL-10 (Fig 4E) and IL-17A (Fig 4F) between WT and *Ncf1*^*-/-*^ mice. We also assessed the levels of several neutrophil chemokines. Increased levels of chemokines, including CXCL5, CCL5 and CXCL1, were reported in previous mouse mycobacterial infection experiments [6, 14, 15]. We hence measured these cytokines in the lung tissues. Among these three chemokines, CXCL5, secreted by pulmonary epithelial cells, was previously identified to be a potent neutrophil chemokine which drives an early neutrophilic inflammation in mouse TB infection [6]. We found that all these three chemokines had elevated levels on day 14 when compared with those on day 7 in both groups and significantly higher in the *Ncf1*^*-/-*^ mice when compared with those in the wild type group. It is noteworthy that CXCL1 (KC, the murine IL-8 homologue) level may have peaked in *Ncf1*^*-/-*^ mice on day 7 (Fig 4I), while CCL5 and CXCL5 increased significantly from day 7 to day 14 (Fig 4H). Taken together, our results implicated that the high IL-1β and neutrophilic chemokines including CXCL5, CCL5 and CXCL1 may contribute to the early neutrophilic inflammation in *Ncf1*^*-/-*^ mice.

### Different regulation of IL-1β production by ROS in macrophages and neutrophils

**Fig 5:**
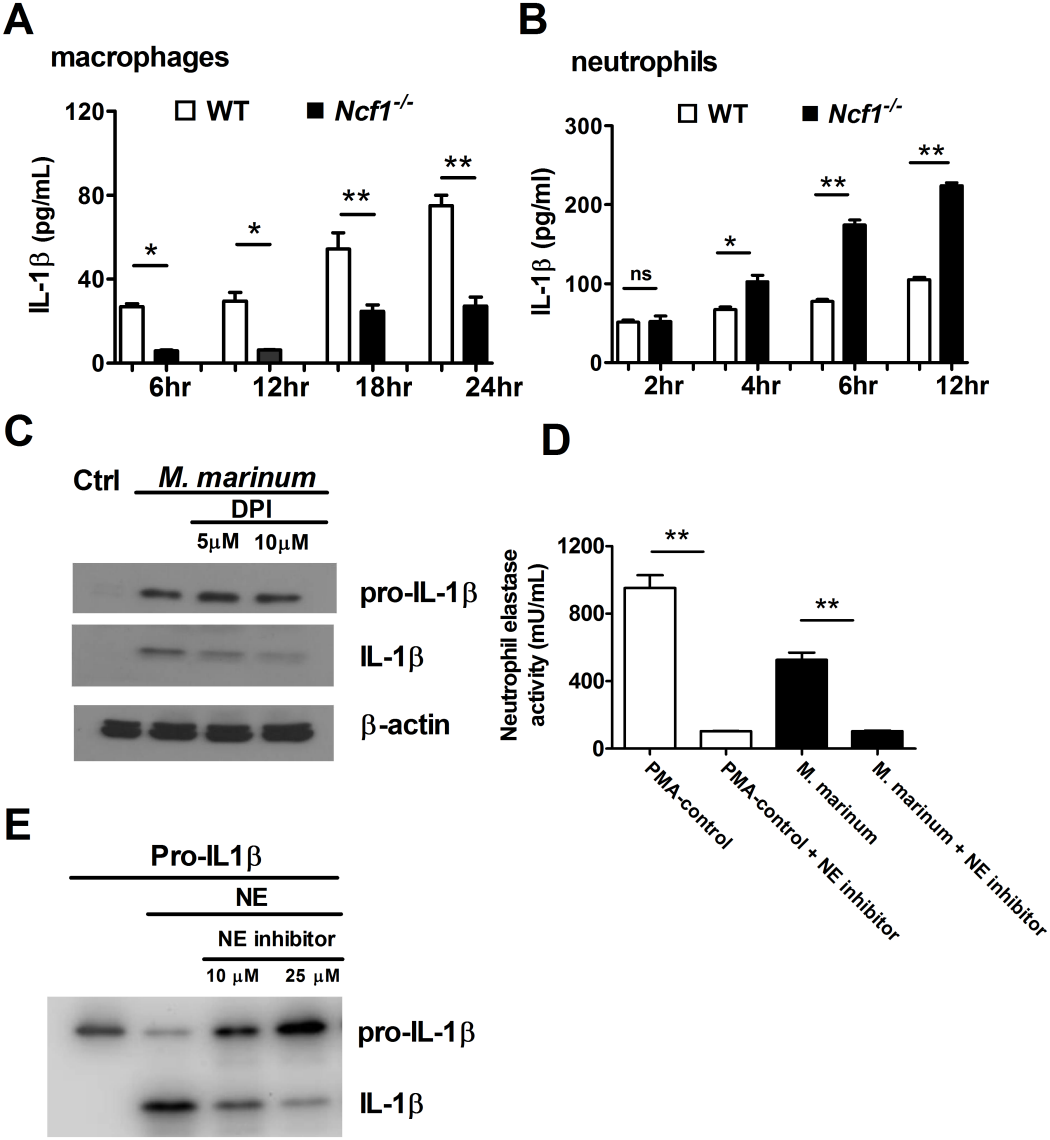
Different IL-1β production in macrophages and neutrophils from *Ncf1*^-/-^ and wild type mice and the activity of neutrophil elastase to process pro-IL-1β. Thioglycollate-elicited macrophages (1x10^5^) and neutrophils (1x10^6^) from *Ncf1*^*-/-*^ and WT mice were infected with *M*. *marinum* at MOI:1, the IL-1β release was then determined by ELISA (A and B). Differentiated THP-1 cells were left uninfected (control) or pretreated by PBS and DPI then infected for 4 h with *M*. *marinum* at MOI:1, 18 h later, pro-IL-1β (p34) and mature-IL-1β (p17) (C) were analyzed by immunoblot. NE activity of human granulocytes (1x10^6^) were left untreated or pre-treated by NE inhibitor and then stimulated with PMA (100nM) as a positive control and *M*. *marinum* (MOI: 5) (D). Recombinant pro-IL1β (50 ng) was treated by NE (2x10^-4^ U) with and without NE inhibitor, and mature-IL-1β (p17) was analyzed by immunoblot (E). Data represent mean ± sd. The experiments were analyzed with Kruskal-Wallis analysis and repeated 2 times with similar results. *p < 0.05; **p < 0.005.

Previous studies, including our own, have indicated that decreased ROS levels lead to a lower inflammasome activation and IL-1β production in mycobacterial infection in macrophages [8, 16, 17]. However, recent studies have shown that neutrophils play an important role in IL-1β production during mycobacterial infection [18, 19]. We hence went on to investigate the role of ROS in regulating IL-1β production in different immune cell populations including macrophages and neutrophils. Using thioglycollate-elicited macrophages and neutrophils of WT and *Ncf1*^*-/-*^ mice, we found that IL-1β production was significantly lower in ROS-deficient macrophages (Fig 5A), whereas the IL-1β production tended to be higher in ROS-deficient neutrophils (Fig 5B) after *M*. *marinum* infection. To clarify the relationship between ROS and pro-IL-1β cleavage in macrophages, we investigated the effect of ROS on the IL-1β production in a monocytic cell line. The western blot analysis of pro-IL-1β and IL-1β in THP1 cells stimulated with *M*. *marinum* in the presence or absence of the NOX2 inhibitor diphenyleneiodonium (DPI) showed that ROS production facilitates the cleavage of pro-IL-1β to IL-1β in monocytic cells (Fig 5C).

### Neutrophil elastase is active in processing pro-IL-1β

Based on the abundant infiltration of NE-positive neutrophils in *M*. *marinum*-infected *Ncf1*^*-/-*^ mice, we then tested the protease activity of NE, a neutrophil-specific protease, in activating pro-IL-1β. We tested the activity of NE in granulocytes and found a high NE activity, which can be inhibited by an enzyme inhibitor, in granulocytes after *M*. *marinum* infection (Fig 5D). We then specifically tested the activity of NE to process pro-IL-1β. We found that NE was able to process pro-IL-1β to produce IL-1β and this protease activity was reduced by adding inhibitors of NE in a dose-response manner (Fig 5E). These data suggested that the elevated *in vivo* levels of active IL-1β in the lung of *M*. *marinum*-infected *Ncf1*^*-/-*^ mice may result from the cleavage of pro- IL-1β by NE from the neutrophils in the tissue.

### Expression of neutrophil elastase and IL-1β in the caseous granulomatous inflammation of human pulmonary tuberculosis

**Fig 6:**
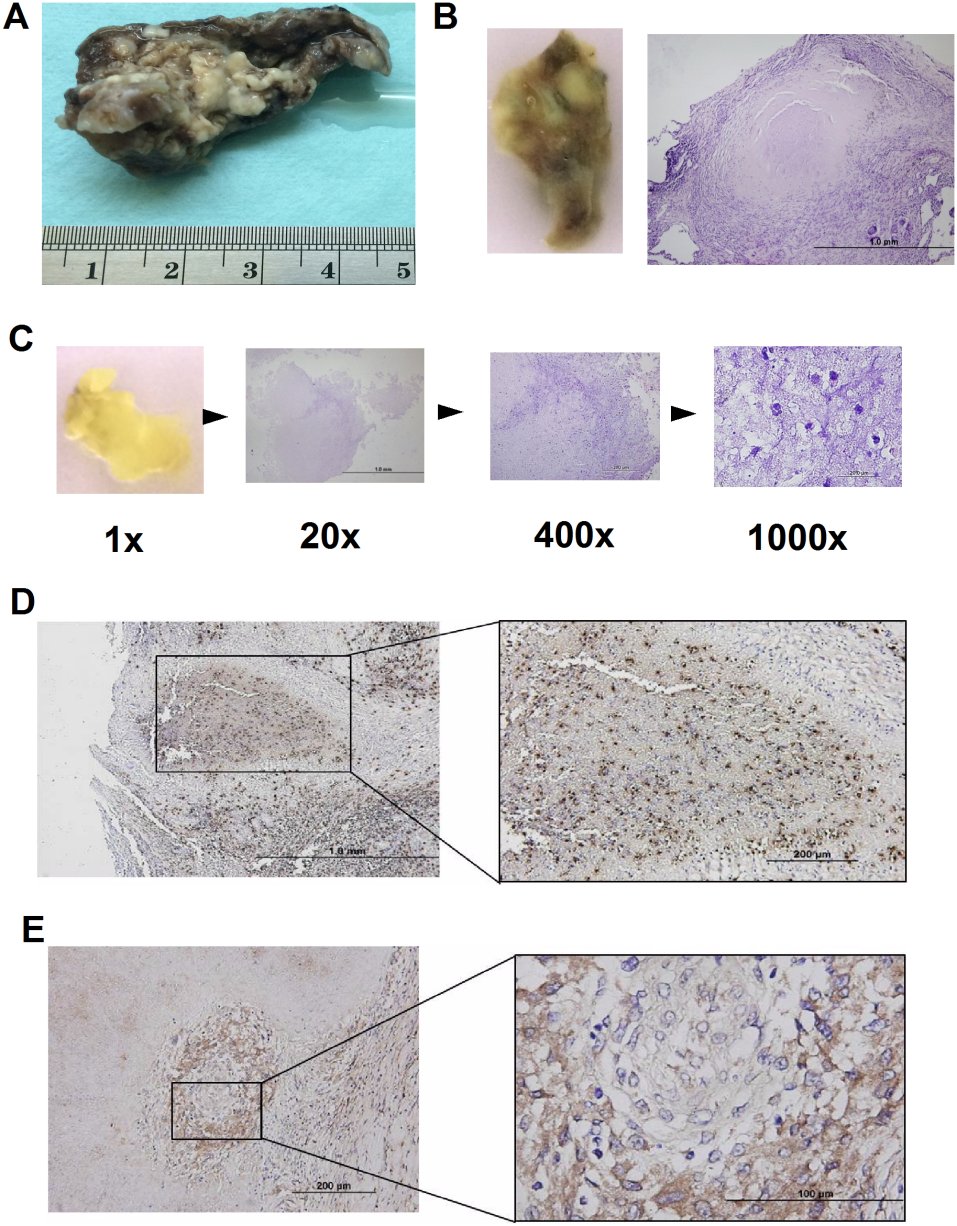
Expression of neutrophil elastase and IL-1β in the caseous granulomatous inflammation in the human lung tissues of pulmonary tuberculosis. Formalinized lung tissue (A) of one patient with TB infection. The solid part (B) of the lung tissue showed characteristic caseous granulomatous inflammation with Langhans giant cells, and numerous neutrophils were found within the caseous part (C) of the lung tissues in high power field tissue. Immunohistochemical stain of neutrophil elastase (D) and IL-1β (E) showed abundant neutrophil elastase-positive cells and IL-1β in the granulomatous inflammation.

To extend our findings from mouse to human and from *M*. *marinum* to *M*. *tuberculosis*, we examined lung tissue samples from patients with pulmonary tuberculosis (Fig 6A). Characteristic caseous granulomatous inflammation with Langhans giant cells was clearly demonstrated by histological examination (Fig 6B). In line with one previous report [3], numerous neutrophils were found within the caseous tissue from the pulmonary cavity resulting from TB infection (Fig 6C). Importantly, the immunohistochemical staining with anti-NE antibody showed abundant NE-positive cell within the caseous area (Fig 6D). Furthermore, the immunohistochemical stain of IL-1β showed many IL-1β-positive cells in caseous granulomatous inflammation (Fig 6E). These data provide evidence supporting the potential roles of NE and IL-1β in human TB infection.

### Blocking of IL-1β signaling alleviates the pulmonary neutrophilic inflammation induced by mycobacterial infection

**Fig 7:**
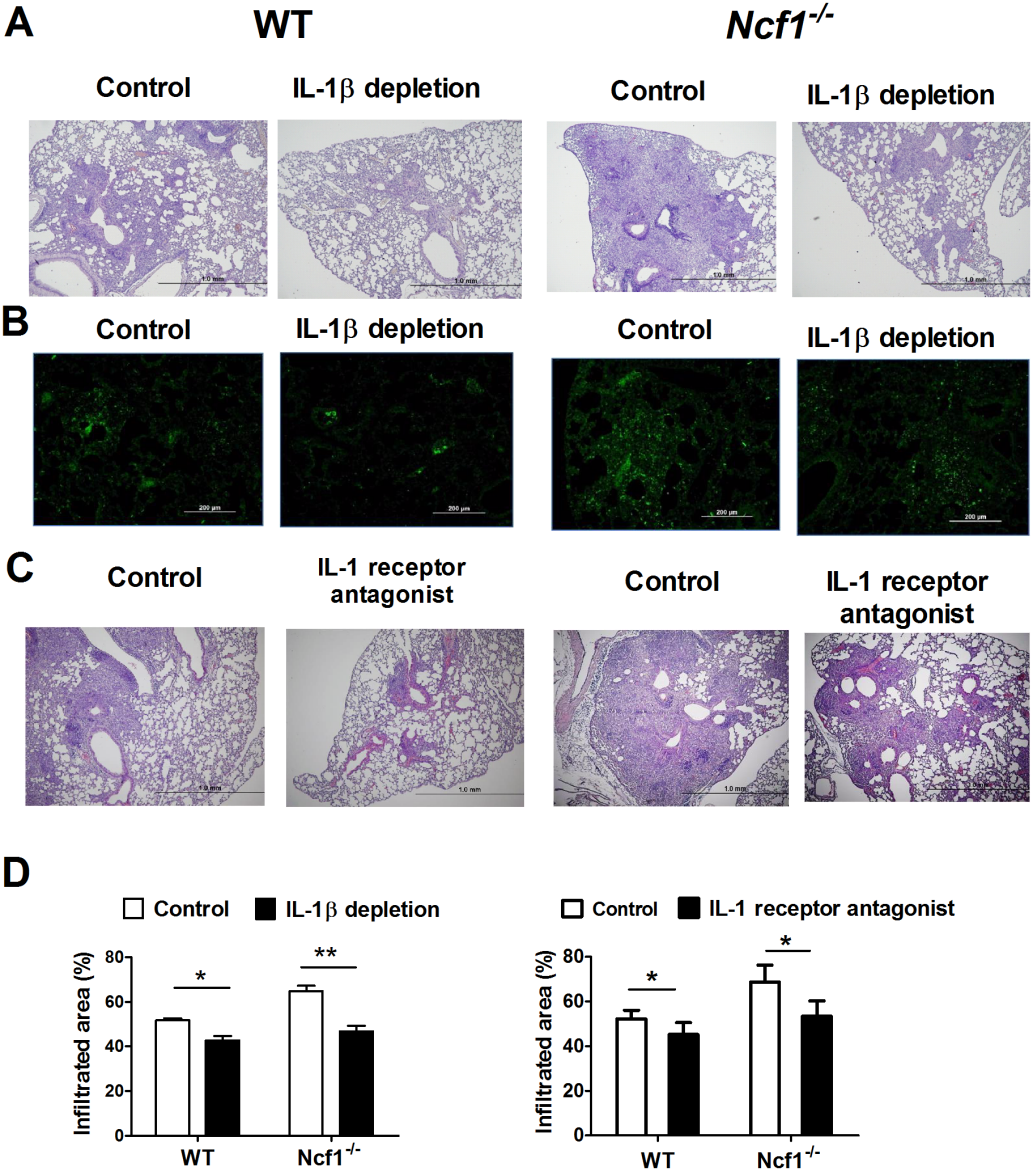
IL-1β depletion and IL-1 receptor antagonist treatment alleviated the neutrophilic inflammation. The characteristic pulmonary histopathological images (A) of *M*. *marinum*-infected *Ncf1*^*-/-*^ mice and WT mice on day-5 with and without IL-1β depletion by a monoclonal antibody directed against IL-1β (150μg on day-1). The immunofluorescent stain of neutrophil elastase (NE) (B) also showed an apparently decrease of NE-positive cells after the depletion of IL-1β. The representative histological images (C) of WT and *Ncf1*^-/-^mice treated with and without IL-1 receptor antagonist (Anakinra, 100mg/kg/day for 5days). Percentages of infiltration area (D) were quantified by using Image J. The experiments were analyzed with Kruskal-Wallis test and repeated with similar results. *p < 0.05; **p < 0.005.

To define the role of IL-1β in this mycobacterial-induced pulmonary neutrophilic inflammation, we used both a monoclonal antibody to deplete IL-1β and a commercial IL-1 receptor antagonist (Anakinra, Kineret^®^). We found that depletion of IL-1β ameliorated the tissue inflammation in both *M*. *marinum*-infected *Ncf1*^*-/-*^ and WT mice on day5 after infection (Fig 7A). The immunofluorescent stain of NE also demonstrated a marked decrease in NE-positive cells after depletion of IL-1β (Fig 7B). Similarly, after the treatment with IL-1 receptor antagonist, the histological changes of pulmonary inflammation were attenuated (Fig 7C). The quantification of infiltration area showed that blocking of IL-1 signaling reduced the mycobacterium-induced pulmonary inflammation in WT and *Ncf1*^*-/-*^ mice (Fig 7D). Collectively, these data suggested that early influx of neutrophils in *M*. *marinum*-infected *Ncf1*^-/-^ mice may potentially contribute to the IL-1β production through NE. Blocking of IL-1β may therefore ameliorate this neutrophilic inflammation in mycobacterial pulmonary infection.

## Discussion

Dysregulated inflammation induced by mycobacterial infection leads to host-detrimental tissue reactions including excessive lung infiltration and cavitary lesions in the lung. In this study, we showed that ROS-deficiency leads to an early and extensive neutrophilic inflammation, which may contribute to the elevated IL-1β production in mycobacterial infection. These findings reveal a novel role of ROS in the early neutrophilic granulomatous inflammation and regulation of IL-1β production in mycobacterial infection.

We used *M*. *marinum* as a surrogate microorganism of *M*. *tb* to investigate mycobacterium-related granulomatous and IL-1 pathway. Although not a common pathogen in the human lung, *M*. *marinum*, a mycobacterium containing RD-1, which is a critical virulence generation of *M*. *tb*, induces cutaneous suppurative granulomatous inflammation in infected humans [20, 21]. *M*. *marinum*-induced reaction bears marked histological characteristics similar to granulomas induced by *M*. *tb* in the lung and has been increasingly used in mechanistic studies of mycobacteria-induced inflammation [22]. Additionally, Cooper et al. found a higher mycobacterial load, a higher level of IFN-γ, and more severe pulmonary inflammation with aggregates of neutrophils after *M*. *tb* infection in *Ncf1*^*-/-*^ mice than those in WT mice [23]. In this study, to minimize the effects of mycobacterial load on cytokines production, we decreased *M*. *marinum* to 3 x10^6^ in experiments studying the effects of ROS on the production of cytokines and chemokines (Fig 4). In the low-dose infection, we found a similar CFU counts between WT mice and *Ncf1*^*-/-*^ mice (S1 Fig). The differences in the cytokine levels we observed in this study hence should be attributed to the distinct tissue immunoregulatory conditions rather than the bacterial loads. In human TB infection, as demonstrated in a non-human primate model (macaques) with low-dose *M*.*tb* (25 CFU) by using bronchoscopic injection, 5–6 weeks is required to find characteristic pulmonary inflammation after the infection [24]. However, such precise low-dose infection and long follow-up duration have difficulty to conduct in animals other than non-human primate. High-dose mycobacterial infection protocols hence were generally used in mouse models, including this study, which take only few days to find prominent pulmonary inflammation after infection [6, 25]. This limitation in the mouse models may partly explain the differences in the findings from mouse mycobacterial infection models and human mycobacterial diseases.

The formation of granulomas, organized aggregates of immune cells, is a hallmark microscopic finding of mycobacterial infection in tissues [26]. Recent studies have shown that granulomas are highly dynamic structures containing neutrophils, macrophages, lymphocytes and other cell types [27, 28]. Early influx of neutrophils may critically affect death pattern of infected cells and the subsequent formation of granulomas, which have been reported to be a significant determining factor for unfavorable clinical outcomes including latent pulmonary TB and advanced pulmonary TB with cavity formation [2, 29, 30]. The host-detrimental role of neutrophilic inflammation in TB infection was previously shown in IFN-γ-deficient mice by Nandi et al. [31]. In ROS-deficient mice, we found a marked increase of CXCL5 (Fig 4G), a pulmonary epithelial cell-produced chemokine which may contribute to the recruitment of neutrophils early in mycobacterial infection [6]. The loose *M*. *marinum*-induced neutrophilic granulomas in ROS-deficient mice shown in our results (Fig 3A and 3B) are similar to the recent report by Deffert et al., showing less compact neutrophilic granulomas in mycobacterium-infected mice with defective NOX2 [25]. In that study, they also found that *M*. *bovis* BCG (Bacillus Calmette Guérin) infection induced more severe lung damage in NOX2-deficient mice. They further demonstrated that restoring NOX2 function in macrophages/dendritic cells, but not in neutrophils, led to a marked decrease in the lung damage, indicating different roles of ROS in macrophages and neutrophils during mycobacterial infection [25]. Those findings suggest that early influx of neutrophils may lead to loose granulomas, which in turn result in defective mycobacterial sequestration and the poor control of mycobacterial growth.

In this study, we used RD-1 containing *M*. *marinum* and found that the difference in inflammasome-dependent and inflammasome-independent production of IL-1β in neutrophils and macrophage may be a critical factor underlying the differences between wild type and NOX2-deficient mice. A number of studies have shown the key role of IL-1β in controlling mycobacterial growth [9, 32, 33]. However, the specific role of IL-1β in the host-detrimental tissue inflammation during mycobacterial infection remains unclear due to the difficulty in comparing the severity of granulomatous inflammation without equalizing mycobacterial loads in different mouse strains. Mishra et al. used a streptomycin-dependent infection model to control the growth of *M*. *tb* and found that, given similar mycobacterial load, high levels of IL-1β led to exacerbated pathology including less compact neutrophilic granulomatous inflammation and high mortality in TB infection [34]. IL-1β is a critical innate cytokine in mycobacterial infection and has complex cross-talk with other mediators within the complex inflammatory network consisting of IL-1β, TNF-α, eicosanoids, chemokines, and Th17 response [4, 35]. Therefore, the balance among these innate immune pro-inflammatory cytokines is crucial, and the interaction between cytokine networks and cell death patterns may in turn orchestrate the inflammation in mycobacterial infection [36]. Moreover, the unbalanced pro-inflammatory cytokines also leads to an early influx of neutrophils, which may impede the mycobactericidal ability of resident macrophages, so-called Trojan Horse phenomenon [30]. Along the same line of high IL-1β levels found in *Ncf1*^*-/-*^ mice reported in this study, those results underscore the importance of the redox regulation of the acute inflammation cytokine IL-1β in mycobacterial infection in the lung.

Neutrophil-derived proteases, including NE or cathepsins, have been implicated in caspase-1 independent pro-IL-1β processing in arthritis and *Pseudomonas aeruginosa* infection mouse models [37, 38]. It is now well recognized that inflammasomes are not the only mechanism for processing IL-1 cytokines. High IL-1β production and high mortality were unexpectedly found in *M*. *tb*-infected mice deficient in caspase-1 implicates the existence of caspase-1 independent IL-1β production in mycobacterial infection [39]. Similarly, our laboratory used a serum-induced arthritis mouse model to identify cathepsin B as one of the major proteases in the pro-IL-1β processing in the ROS-deficient *Ncf1*^-/-^ mice [12]. By immunohistochemical analysis of the human lung with TB infection, we found abundant NE, but almost no cathepsin staining in the inflammatory tissue. We therefore inferred that NE may be capable of processing pro-IL-1β to IL-1β in the lung with TB infection. Indeed, we found that neutrophils, which are more abundant in the *Ncf1*^*-/-*^ mice with mycobacterial infection, produced more IL-1β and the protease NE in neutrophils was capable of processing pro-IL-1β (Fig 5).

Modulating the death patterns of inflammatory cells is one of the evasion mechanisms used by *M*. *tb*. to avoid immune clearance. These include regulation of the eicosanoid pathways in macrophages and the programmed death-1 (PD-1) pathway in dendritic cells [29, 40]. Divangahi et al. reported that a high prostaglandin E2 (PGE2) level in macrophages is crucial for the initiation of adaptive immunity. Moreover, Periasamy et al. found that PD-1 pathway orchestrates the expansion of regulatory T cell in *M*. *tb* infection [36, 41]. Our previous study also found that inhibition of ROS regulates the production of PGE2 in macrophage [8]. However, we found comparable levels of adaptive immunity cytokines including IL-10 and IL-17A between WT and *Ncf1*^*-/-*^ mice after *M*. *marinum* infection in this study (Fig 4e and 4f). We hence postulated that, instead of affecting Treg and Th17 pathway, ROS appears to be mainly involved in the early phase of mycobacterial infection and leads to a dysregulated lung inflammation.

Our findings suggest that inhibition of inflammasome alone may be insufficient to suppress IL-1β-related inflammation due to the relative importance of inflammasome-independent pathways in the lungs infected by mycobacteria. The reduced inflammation by abrogation of the IL-1β signal as we showed in this study (Fig 7) and clinical evidence suggesting that IL-1 blockade is relatively safe in patients with rheumatic disorders regarding tuberculosis risk [42] indicate the potential application of IL-1 blockade as an adjunctive therapy to ameliorate tissue inflammation, termed host-directed therapy, in TB infection in combination with the control of mycobacterial growth with conventional anti-TB antibiotic treatment [43]. In the groups of TB patients with impaired leukocyte ROS production, including the patients with diabetes mellitus, higher disease severity and poorer treatment outcome have been attributed to high levels of pro-inflammatory cytokines, including IL-1β, in the tissue [8, 44, 45]. Our results hence suggest that IL-1 blocking may ameliorate inflammation-induced tissue damage and improve the treatment outcome especially in those groups of patients.

In conclusion, we found that lack of ROS leads to the early influx of neutrophils in the formation of granulomas, which in turn leads to exacerbated inflammation. In addition to showing that NOX2 deficient neutrophils are effective cells in inflammasome-independent cleavage of pro-IL-1β, our results suggest the potential use of IL-1β-blocking agents as adjunctive therapies with antimicrobial agents in TB treatment. Our findings shed light on the redox regulatory mechanism of granulomatous inflammation in mycobacterial infection and may contribute to the identification of molecular targets for optimal treatment for TB infection in the future.

## Supporting information

S1 Dataset (XLSX)

S1 Fig Low-dose mycobacterial infection led to similar bacterial loads in *Ncf1*^*-/-*^ and WT mice. (PDF)

## References

[1] World Health Organization. Global tuberculosis report, 2016. p. 1–214. http://www.who.int/tb/publications/global_report/en/

[2] Cadena AM, Flynn JL, Fortune SM. The Importance of First Impressions: Early Events in Mycobacterium tuberculosis Infection Influence Outcome. mBio. 2016;7(2). doi: 10.1128/mBio.00342-16 . 27048801 10.1128/mBio.00342-16 PMC4817258

[3] Eum SY, Kong JH, Hong MS, Lee YJ, Kim JH, Hwang SH, et al Neutrophils are the predominant infected phagocytic cells in the airways of patients with active pulmonary TB. Chest. 2010;137(1):122–8. doi: 10.1378/chest.09-0903 . 19749004 10.1378/chest.09-0903 PMC2803122

[4] Kaufmann SH, Dorhoi A. Inflammation in tuberculosis: interactions, imbalances and interventions. Current opinion in immunology. 2013;25(4):441–9. doi: 10.1016/j.coi.2013.05.005 . 23725875 10.1016/j.coi.2013.05.005

[5] Dorhoi A, Iannaccone M, Maertzdorf J, Nouailles G, Weiner J 3rd, Kaufmann SH. Reverse translation in tuberculosis: neutrophils provide clues for understanding development of active disease. Frontiers in immunology. 2014;5:36 doi: 10.3389/fimmu.2014.00036 . 24550920 10.3389/fimmu.2014.00036 PMC3913996

[6] Nouailles G, Dorhoi A, Koch M, Zerrahn J, Weiner J 3rd, Fae KC, et al CXCL5-secreting pulmonary epithelial cells drive destructive neutrophilic inflammation in tuberculosis. The Journal of clinical investigation. 2014;124(3):1268–82. doi: 10.1172/JCI72030 . 24509076 10.1172/JCI72030 PMC3934185

[7] Marakalala MJ, Raju RM, Sharma K, Zhang YJ, Eugenin EA, Prideaux B, et al Inflammatory signaling in human tuberculosis granulomas is spatially organized. Nature medicine. 2016 doi: 10.1038/nm.4073 . 27043495 10.1038/nm.4073 PMC4860068

[8] Chao WC, Yen CL, Wu YH, Chen SY, Hsieh CY, Chang TC, et al Increased resistin may suppress reactive oxygen species production and inflammasome activation in type 2 diabetic patients with pulmonary tuberculosis infection. Microbes and infection / Institut Pasteur. 2015;17(3):195–204. doi: 10.1016/j.micinf.2014.11.009 . 25528597 10.1016/j.micinf.2014.11.009

[9] Jayaraman P, Sada-Ovalle I, Nishimura T, Anderson AC, Kuchroo VK, Remold HG, et al IL-1beta promotes antimicrobial immunity in macrophages by regulating TNFR signaling and caspase-3 activation. Journal of immunology. 2013;190(8):4196–204. doi: 10.4049/jimmunol.1202688 . 23487424 10.4049/jimmunol.1202688 PMC3622150

[10] Di Paolo NC, Shafiani S, Day T, Papayannoupoulou T, Russell DW, Iwakura Y, et al Interdependence between Interleukin-1 and Tumor Necrosis Factor Regulates TNF-Dependent Control of Mycobacterium tuberculosis Infection. Immunity. 2015;43(6):1125–36. doi: 10.1016/j.immuni.2015.11.016 . 26682985 10.1016/j.immuni.2015.11.016 PMC4685953

[11] Mayer-Barber KD, Andrade BB, Oland SD, Amaral EP, Barber DL, Gonzales J, et al Host-directed therapy of tuberculosis based on interleukin-1 and type I interferon crosstalk. Nature. 2014;511(7507):99–103. doi: 10.1038/nature13489 . 24990750 10.1038/nature13489 PMC4809146

[12] Huang YF, Lo PC, Yen CL, Nigrovic PA, Chao WC, Wang WZ, et al Redox Regulation of Pro-IL-1beta Processing May Contribute to the Increased Severity of Serum-Induced Arthritis in NOX2-Deficient Mice. Antioxidants & redox signaling. 2015;23(12):973–84. doi: 10.1089/ars.2014.6136 . 25867281 10.1089/ars.2014.6136 PMC4624247

[13] Marzo E, Vilaplana C, Tapia G, Diaz J, Garcia V, Cardona PJ. Damaging role of neutrophilic infiltration in a mouse model of progressive tuberculosis. Tuberculosis (Edinb). 2014;94(1):55–64. doi: 10.1016/j.tube.2013.09.004 . 24291066 10.1016/j.tube.2013.09.004

[14] Chtanova T, Schaeffer M, Han SJ, van Dooren GG, Nollmann M, Herzmark P, et al Dynamics of neutrophil migration in lymph nodes during infection. Immunity. 2008;29(3):487–96. doi: 10.1016/j.immuni.2008.07.012 . 18718768 10.1016/j.immuni.2008.07.012 PMC2569002

[15] Vesosky B, Rottinghaus EK, Stromberg P, Turner J, Beamer G. CCL5 participates in early protection against Mycobacterium tuberculosis. J Leukoc Biol. 2010;87(6):1153–65. doi: 10.1189/jlb.1109742 . 20371596 10.1189/jlb.1109742 PMC2872537

[16] Martinon F. Signaling by ROS drives inflammasome activation. Eur J Immunol. 2010;40(3):616–9. Epub 2010/03/05. doi: 10.1002/eji.200940168 . 20201014 10.1002/eji.200940168

[17] Chen CC, Tsai SH, Lu CC, Hu ST, Wu TS, Huang TT, et al Activation of an NLRP3 inflammasome restricts Mycobacterium kansasii infection. PLoS One. 2012;7(4):e36292 Epub 2012/05/05. doi: 10.1371/journal.pone.0036292 . 22558425 10.1371/journal.pone.0036292 PMC3340363

[18] Cho JS, Guo Y, Ramos RI, Hebroni F, Plaisier SB, Xuan C, et al Neutrophil-derived IL-1beta is sufficient for abscess formation in immunity against Staphylococcus aureus in mice. PLoS pathogens. 2012;8(11):e1003047 doi: 10.1371/journal.ppat.1003047 . 23209417 10.1371/journal.ppat.1003047 PMC3510260

[19] Mohammadi N, Midiri A, Mancuso G, Patane F, Venza M, Venza I, et al Neutrophils Directly Recognize Group B Streptococci and Contribute to Interleukin-1beta Production during Infection. PLoS One. 2016;11(8):e0160249 doi: 10.1371/journal.pone.0160249 . 27509078 10.1371/journal.pone.0160249 PMC4980021

[20] Stinear TP, Seemann T, Harrison PF, Jenkin GA, Davies JK, Johnson PD, et al Insights from the complete genome sequence of Mycobacterium marinum on the evolution of Mycobacterium tuberculosis. Genome Res. 2008;18(5):729–41. doi: 10.1101/gr.075069.107 . 18403782 10.1101/gr.075069.107 PMC2336800

[21] Travis WD, Travis LB, Roberts GD, Su DW, Weiland LW. The histopathologic spectrum in Mycobacterium marinum infection. Archives of pathology & laboratory medicine. 1985;109(12):1109–13. . 3840985

[22] Myllymaki H, Bauerlein CA, Ramet M. The Zebrafish Breathes New Life into the Study of Tuberculosis. Frontiers in immunology. 2016;7:196 doi: 10.3389/fimmu.2016.00196 . 27242801 10.3389/fimmu.2016.00196 PMC4871865

[23] Cooper AM, Segal BH, Frank AA, Holland SM, Orme IM. Transient loss of resistance to pulmonary tuberculosis in p47(phox-/-) mice. Infection and immunity. 2000;68(3):1231–4. . 10678931 10.1128/iai.68.3.1231-1234.2000 PMC97272

[24] Lin PL, Pawar S, Myers A, Pegu A, Fuhrman C, Reinhart TA, et al Early events in Mycobacterium tuberculosis infection in cynomolgus macaques. Infection and immunity. 2006;74(7):3790–803. doi: 10.1128/IAI.00064-06 . 16790751 10.1128/IAI.00064-06 PMC1489679

[25] Deffert C, Schappi MG, Pache JC, Cachat J, Vesin D, Bisig R, et al Bacillus calmette-guerin infection in NADPH oxidase deficiency: defective mycobacterial sequestration and granuloma formation. PLoS pathogens. 2014;10(9):e1004325 doi: 10.1371/journal.ppat.1004325 . 25188296 10.1371/journal.ppat.1004325 PMC4154868

[26] Williams GT, Williams WJ. Granulomatous inflammation—a review. Journal of clinical pathology. 1983;36(7):723–33. . 6345591 10.1136/jcp.36.7.723 PMC498378

[27] Davis JM, Ramakrishnan L. The role of the granuloma in expansion and dissemination of early tuberculous infection. Cell. 2009;136(1):37–49. doi: 10.1016/j.cell.2008.11.014 . 19135887 10.1016/j.cell.2008.11.014 PMC3134310

[28] Dorhoi A, Kaufmann SH. Pathology and immune reactivity: understanding multidimensionality in pulmonary tuberculosis. Seminars in immunopathology. 2016;38(2):153–66. doi: 10.1007/s00281-015-0531-3 . 26438324 10.1007/s00281-015-0531-3

[29] Divangahi M, Behar SM, Remold H. Dying to live: how the death modality of the infected macrophage affects immunity to tuberculosis. Advances in experimental medicine and biology. 2013;783:103–20. Epub 2013/03/08. doi: 10.1007/978-1-4614-6111-1_6 . 23468106 10.1007/978-1-4614-6111-1_6 PMC4678885

[30] Warren E, Teskey G, Venketaraman V. Effector Mechanisms of Neutrophils within the Innate Immune System in Response to Mycobacterium tuberculosis Infection. Journal of clinical medicine. 2017;6(2). doi: 10.3390/jcm6020015 . 28178208 10.3390/jcm6020015 PMC5332919

[31] Nandi B, Behar SM. Regulation of neutrophils by interferon-gamma limits lung inflammation during tuberculosis infection. The Journal of experimental medicine. 2011;208(11):2251–62. doi: 10.1084/jem.20110919 . 21967766 10.1084/jem.20110919 PMC3201199

[32] Juffermans NP, Florquin S, Camoglio L, Verbon A, Kolk AH, Speelman P, et al Interleukin-1 signaling is essential for host defense during murine pulmonary tuberculosis. The Journal of infectious diseases. 2000;182(3):902–8. doi: 10.1086/315771 . 10950787 10.1086/315771

[33] Di Paolo NC, Shafiani S, Day T, Papayannopoulou T, Russell DW, Iwakura Y, et al Interdependence between Interleukin-1 and Tumor Necrosis Factor Regulates TNF-Dependent Control of Mycobacterium tuberculosis Infection. Immunity. 2015;43(6):1125–36. doi: 10.1016/j.immuni.2015.11.016 . 26682985 10.1016/j.immuni.2015.11.016 PMC4685953

[34] Mishra BB, Rathinam VA, Martens GW, Martinot AJ, Kornfeld H, Fitzgerald KA, et al Nitric oxide controls the immunopathology of tuberculosis by inhibiting NLRP3 inflammasome-dependent processing of IL-1beta. Nature immunology. 2013;14(1):52–60. doi: 10.1038/ni.2474 . 23160153 10.1038/ni.2474 PMC3721324

[35] Stephen-Victor E, Sharma VK, Das M, Karnam A, Saha C, Lecerf M, et al IL-1beta, But Not Programed Death-1 and Programed Death Ligand Pathway, Is Critical for the Human Th17 Response to Mycobacterium tuberculosis. Frontiers in immunology. 2016;7:465 doi: 10.3389/fimmu.2016.00465 . 27867382 10.3389/fimmu.2016.00465 PMC5095489

[36] Divangahi M, Desjardins D, Nunes-Alves C, Remold HG, Behar SM. Eicosanoid pathways regulate adaptive immunity to Mycobacterium tuberculosis. Nature immunology. 2010;11(8):751–8. Epub 2010/07/14. doi: 10.1038/ni.1904 . 20622882 10.1038/ni.1904 PMC3150169

[37] Joosten LA, Netea MG, Fantuzzi G, Koenders MI, Helsen MM, Sparrer H, et al Inflammatory arthritis in caspase 1 gene-deficient mice: contribution of proteinase 3 to caspase 1-independent production of bioactive interleukin-1beta. Arthritis and rheumatism. 2009;60(12):3651–62. doi: 10.1002/art.25006 . 19950280 10.1002/art.25006 PMC2993325

[38] Karmakar M, Sun Y, Hise AG, Rietsch A, Pearlman E. Cutting edge: IL-1beta processing during Pseudomonas aeruginosa infection is mediated by neutrophil serine proteases and is independent of NLRC4 and caspase-1. Journal of immunology. 2012;189(9):4231–5. doi: 10.4049/jimmunol.1201447 . 23024281 10.4049/jimmunol.1201447 PMC3482477

[39] Mayer-Barber KD, Barber DL, Shenderov K, White SD, Wilson MS, Cheever A, et al Caspase-1 independent IL-1beta production is critical for host resistance to mycobacterium tuberculosis and does not require TLR signaling in vivo. Journal of immunology. 2010;184(7):3326–30. doi: 10.4049/jimmunol.0904189 . 20200276 10.4049/jimmunol.0904189 PMC3420351

[40] Stephen-Victor E, Saha C, Sharma M, Holla S, Balaji KN, Kaveri SV, et al Inhibition of programmed death 1 ligand 1 on dendritic cells enhances Mycobacterium-mediated interferon gamma (IFN-gamma) production without modulating the frequencies of IFN-gamma-producing CD4+ T cells. The Journal of infectious diseases. 2015;211(6):1027–9. doi: 10.1093/infdis/jiu532 . 25258379 10.1093/infdis/jiu532

[41] Periasamy S, Dhiman R, Barnes PF, Paidipally P, Tvinnereim A, Bandaru A, et al Programmed death 1 and cytokine inducible SH2-containing protein dependent expansion of regulatory T cells upon stimulation With Mycobacterium tuberculosis. The Journal of infectious diseases. 2011;203(9):1256–63. doi: 10.1093/infdis/jir011 . 21383382 10.1093/infdis/jir011 PMC3069733

[42] Cantini F, Niccoli L, Goletti D. Tuberculosis risk in patients treated with non-anti-tumor necrosis factor-alpha (TNF-alpha) targeted biologics and recently licensed TNF-alpha inhibitors: data from clinical trials and national registries. The Journal of rheumatology Supplement. 2014;91:56–64. doi: 10.3899/jrheum.140103 . 24789001 10.3899/jrheum.140103

[43] Wallis RS, Hafner R. Advancing host-directed therapy for tuberculosis. Nature reviews Immunology. 2015;15(4):255–63. doi: 10.1038/nri3813 . 25765201 10.1038/nri3813

[44] Andrade BB, Pavan Kumar N, Sridhar R, Banurekha VV, Jawahar MS, Nutman TB, et al Heightened plasma levels of heme oxygenase-1 and tissue inhibitor of metalloproteinase-4 as well as elevated peripheral neutrophil counts are associated with TB-diabetes comorbidity. Chest. 2014;145(6):1244–54. doi: 10.1378/chest.13-1799 . 24458266 10.1378/chest.13-1799 PMC4042512

[45] Kumar NP, Sridhar R, Banurekha VV, Jawahar MS, Fay MP, Nutman TB, et al Type 2 diabetes mellitus coincident with pulmonary tuberculosis is associated with heightened systemic type 1, type 17, and other proinflammatory cytokines. Ann Am Thorac Soc. 2013;10(5):441–9. doi: 10.1513/AnnalsATS.201305-112OC . 23987505 10.1513/AnnalsATS.201305-112OC PMC3960913

