# Supplementary material for: Mycobacterial infection induces higher interleukin-1β and dysregulated lung inflammation in mice with defective leukocyte NADPH oxidase
Source: PLoS One. 2017 Dec 11;12(12):e0189453. doi: 10.1371/journal.pone.0189453 (PMC5724816; doi:10.1371/journal.pone.0189453)
Supplement: S1 Fig — (PDF) [file pone.0189453.s002.pdf]

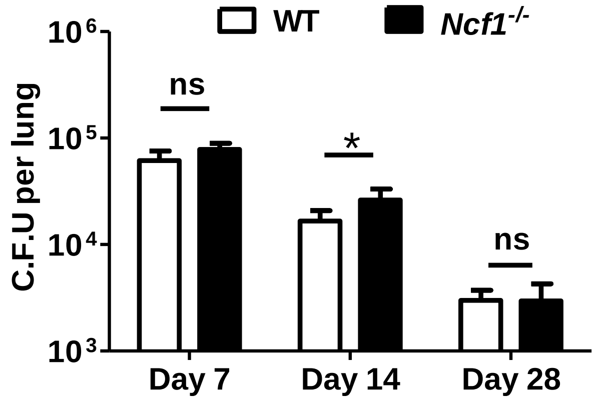

S1 Fig Low-dose mycobacterial infection led to similar bacterial loads in *Ncf1*$^{-/-}$and WT mice.

Ncf1$^{-/-}$ and WT controls were intra-tracheal injected with *M. marinum* (3 x 10^6 CFU). The number of viable mycobacteria was determined at 7 days, 14 days and 28 days after *M. marinum* infection. Data are shown as amean log of CFU per paired-lung (5 mice per group).
